# Representative transcript sets for evaluating a translational initiation sites predictor

**DOI:** 10.1186/1471-2105-10-206

**Published:** 2009-07-02

**Authors:** Jia Zeng, Reda Alhajj, Douglas J Demetrick

**Affiliations:** 1 Department of Computer Science, University of Calgary, Calgary, AB, T2N 1N4, Canada; 2 Department of Computer Science, Global University, Beirut, Lebanon; 3 Department of Pathology & Laboratory Medicine, Oncology, Biochemistry & Molecular Biology, University of Calgary, Calgary, AB, T2N 1N4, Canada

## Abstract

**Background:**

Translational initiation site (TIS) prediction is a very important and actively studied topic in bioinformatics. In order to complete a comparative analysis, it is desirable to have several benchmark data sets which can be used to test the effectiveness of different algorithms. An ideal benchmark data set should be reliable, representative and readily available. Preferably, proteins encoded by members of the data set should also be representative of the protein population actually expressed in cellular specimens.

**Results:**

In this paper, we report a general algorithm for constructing a reliable sequence collection that only includes mRNA sequences whose corresponding protein products present an average profile of the general protein population of a given organism, with respect to three major structural parameters. Four representative transcript collections, each derived from a model organism, have been obtained following the algorithm we propose. Evaluation of these data sets shows that they are reasonable representations of the spectrum of proteins obtained from cellular proteomic studies. Six state-of-the-art predictors have been used to test the usefulness of the construction algorithm that we proposed. Comparative study which reports the predictors' performance on our data set as well as three other existing benchmark collections has demonstrated the actual merits of our data sets as benchmark testing collections.

**Conclusion:**

The proposed data set construction algorithm has demonstrated its property of being a general and widely applicable scheme. Our comparison with published proteomic studies has shown that the expression of our data set of transcripts generates a polypeptide population that is representative of that obtained from evaluation of biological specimens. Our data set thus represents "real world" transcripts that will allow more accurate evaluation of algorithms dedicated to identification of TISs, as well as other translational regulatory motifs within mRNA sequences. The algorithm proposed by us aims at compiling a redundancy-free data set by removing redundant copies of homologous proteins. The existence of such data sets may be useful for conducting statistical analyses of protein sequence-structure relations. At the current stage, our approach's focus is to obtain an "average" protein data set for any particular organism without posing much selection bias. However, with the three major protein structural parameters deeply integrated into the scheme, it would be a trivial task to extend the current method for obtaining a more selective protein data set, which may facilitate the study of some particular protein structure.

## Background

The accurate recognition of *translational initiation sites *(TISs) in mRNA sequences is crucial to identifying the primary structure of the corresponding proteins. A number of computational approaches have been proposed which aim at predicting TISs without human intervention. In order to better evaluate the merit of a newly proposed approach, it is essential to conduct a thorough comparative study that involves several existing approaches. This requires the existence of some high quality benchmark data sets that can be easily used for testing most existing methods.

So far, only a few data sets used for this purpose have been made available and a description of them is in order. Pedersen and Nielsen [1] generated a vertebrate sequence collection and an Arabidopsis thaliana collection. The former consists of sequences from eight model vertebrate organisms and the latter contains sequences from *Arabidopsis thaliana *(thale cress, a dicot plant). The original sequences were the nuclear genes with annotated start codon which were selected from GenBank release 95. Each of them was spliced by eliminating the possible introns and joining the remaining exon parts. Sequences that contain less than 10 nucleotides upstream of the start codon, or less than 150 nucleotides downstream, or have non-nucleotide symbols in the intervals were eliminated. The resulting data sets then underwent a redundancy elimination process which removes redundant sequences that are from homologous genes. The final data sets contain 3312 vertebrates sequences and 523 Arabidopsis thaliana sequences. The vertebrates collection has been widely used in academia as a benchmark collection for a certain TIS recognition method. However, originating from the GenBank, the sequences in this collection are susceptible to sequencing errors. The authors also did not discuss about the quality of the TIS annotation provided by GenBank. As well, due to excessive trimming, on average, a sequence is only about 160 nt long, and it generally does not contain the complete ORF. This may hinder the application of the classifiers which rely on the information about ORFs. Also due to trimming, there exist only about four AUGs in one sequence (within a complete ORF, there are usually around 20 AUGs), which potentially leads to an overly optimistic estimation of the performance of a given algorithm. Both data sets are downloadable from the Internet .

Another existing testing data set was constructed by Nadershahi *et al*. [2], which is also accessible through the public domain ( and ). The authors had compiled a list of 100 human EST sequences, 50 of which contain complete ORFs and 50 do not. Originally a search in the GenBank database for human genomic sequences containing the annotation of "complete CDS" was used. No further discussion regarding the annotation's reliability was given by the authors. A filter for NCBI RefSeq entries was then applied to obtain a non-redundant collection. They were further filtered by using the UniGene clusters as reference. In the end, 50 clusters containing ProtEST links were randomly selected to construct the final data set. Although the authors used this data set to evaluate several state-of-the-art TIS predictors in their paper, it has not been widely used by other researchers, possibly due to the following two factors. Firstly, the collection is fairly small containing only 100 sequences, and secondly, ESTs may include errors leading to frame shifts and they only represent segments of the parent cDNA, therefore require additional curation before the determination of corresponding protein sequence.

Hatzigeorgiou [3] also generated a human cDNA sequence collection to test her neural network TIS classifier. The protein database SwissProt was used as the source, where all the human proteins whose N-terminal sites are sequenced at the amino acid level were collected and manually checked. Then corresponding full-length mRNAs with verified TISs are selected. In the end, 475 derived human cDNAs were obtained. This is one of the few published data sets for TIS prediction that contain highly reliable sequences. However, the collection construction scheme requires human intervention, and the data set is not accessible through the public domain.

Saeys *et al*. [4] compiled a data set from the consensus CDS (CCDS) database by selecting all the genes with a consensus TIS (i.e., the triplet of ATG). The authors' justification of using the CCDS repository is that the core set of human protein coding regions included in the database are consistently annotated and of high quality. Annotation updates represent genes that are defined by a mixture of manual curation and automated computational processing. The quality tests performed include consistency in cross-species analysis, analyses to identify putative pseudogenes, retrotransposed genes, consensus splice sites, supporting transcripts and protein homology. The resulting data collection contains 13917 sequences. There are several limitations of using this collection to test a TIS classifier: *a*) the data set is too large: the file that has undergone compression is almost 40 mega bytes in size, making it quite impractical for efficient testing; *b*) the database itself only contains genomic sequences, which deviates from the type of nucleotide sequences that are actually used by the translational mechanism (mRNAs); and *c*) up to today, CCDS only investigates the core CDS sets of two organisms: human and mouse, which greatly limits the applicability of the collection construction scheme proposed by the authors.

Hu *et al*. [5] proposed a computational approach for predicting TIS in prokaryotic genomes. The method models a *positional weight matrix *(PWM) of aligned sequences around predicted TISs in terms of a linear combination of three elementary PWMs. Based upon the best predictions output by the constituents of the computational model, an annotated TIS database called SupTISA is constructed. The annotation of the data set has been shown to present a high quality when validated on experimentally verified TISs set EcoGene [6], a genome sequence database for *Escherichia coli*. However, there are several drawbacks of this database. Firstly, it is only proposed for prokaryotic genomes, which limits its applicability. Secondly, it is completely built upon an existing TIS predictor, which may involve some selection bias. Therefore, we believe it does not meet the necessary criteria of a benchmark data set.

Cai *et al*. [7] proposed a web-based database called ATID which consists of 300 genes from *Homo sapiens, Mus musculus *and other species, where each gene is shown to have multiple translational initiation sites. In our current version of the algorithm, genes that are associated with alternative translational initiation events are excluded. We believe that the ATID database may serve as a good complement to the data sets generated by our algorithm.

Our review of the literature has inspired us to propose a general scheme for constructing high quality, reliable, representative, non-redundant and easily accessible data sets that can facilitate the evaluation of algorithms used to identify gene characteristics such as TIS prediction. In this paper, we describe such an algorithm. We used four model organisms as an example and reported characteristics on the four sample data sets based on the analysis of the selected proteins' molecular weight, isoelectric point and hydrophobicity profile. These results have led to some interesting observations that may be valuable for protein studies in general. Some open-ended discussion has been provided as well with the hope of inviting further input on the subject from the audience of this article.

## Methods

### Source Sequence Repository

To locate the most reliable sequence source available, we reviewed the following state-of-the-art molecular biology databases. The GenBank is a collection of publicly available annotated nucleotide sequences, including mRNA sequences with coding regions, segments of genomic DNA with a single gene or multiple genes as well as ribosomal RNA gene clusters. Though complete, it includes all sequence data submitted, possibly containing erroneous ones. Therefore the repository is susceptible to redundancy and errors. UniProt is a protein sequence database that was formed through the merger of SwissProt, TrEMBL and PIR-PSD. It provides a collection of functional information on proteins with rich annotation. However the entries in UniProt are represented by amino acid sequences, which cannot be directly mapped to nucleotide sequences. Consequently the database is not suitable for constructing benchmark data sets for TIS predictor evaluation in that almost all of the existing methods only analyze nucleotide sequences. The Reference Sequence (RefSeq) database is a curated collection of DNA, RNA, and protein sequences built by NCBI. It aims to provide a comprehensive, integrated, non-redundant, well-annotated set of sequences for taxonomically diverse organisms ranging from eukaryotes to bacteria to viruses. However, some possible limitations of the annotation procedure applied to yield sequences included in RefSeq are implied in Nielsen and Krogh's study [8] which employs an automatic prokaryotic gene predictor to validate the annotation quality of GenBank and RefSeq respectively using several model prokaryotic organisms. Although we believe that mistakes may exist in some of the RefSeq's annotation, to the best of our knowledge, the sequence repository still ranks higher than its peers in terms of accuracy and reliability. Therefore, we still decide to use RefSeq as the source database to construct the data sets in our experiments. It is worth noting that the algorithm that is to be described in subsequent sections is a general procedure which is independent from the repository that is chosen.

### Implementation

Our data construction algorithm consists of three phases. In Phase I, we start with retrieving a complete mRNA sequence collection for a given organism from the RefSeq database, then we eliminate the sequences that have at least one of the following problems: *a*) the corresponding protein contains non-standard amino acid(s), *b*) the start codon is not AUG, and *c*) there exists some data format error.

Phase II intends to select the most representative mRNA sequences out from the Phase I data set. By representative, we mean the mRNAs whose corresponding protein products are commonly found in the organism proteomics. Three different characteristics are considered in our implemenation: the *molecular weight *(MW), the *isoelectric point *(pI) and the *hydrophobicity index *(HI) profile. Assuming some appropriate settings are found for the parameters of MW and pI, then our approach can identify a dataset that corresponds to a perceived "average" expressed protein population as determined by current proteomic investigations. Due to technical reasons (since analyzing the HI profile is a more computationally intensive task than the previous two), we divide the Phase II selection process into two stages and let us denote the intermediate data collection that include sequences satisfying conditions *a *and *b *by the Phase II-Intermediate collection.

It is a trivial task to compute the molecular weight for a given protein whose primary structure is known. Eq. 1 shows the formula, where *n *refers to the number of amino acids in the protein and *aim*_*i *_refers to the average isotopic mass of the *i*-th amino acid.

(1)

To facilitate the introduction to our solution for calculating pI, let us quickly review the definition of the isoelectric point of a protein – the pH value in which the net charge of the protein is equal to zero. Within this context, the charge of a protein mainly depends on seven charged amino acids: Glu, Asp, Cys, Tyr, His, Lys and Arg, together with the C-terminal and the N-terminal. The Henderson-Hasselbach equation is used to calculate protein charge in a certain pH. Eq. 2 and Eq. 3 display the formulas used to compute negatively charged and positively charged macromolecules respectively.

(2)

(3)

where *pK*_*n *_is the acid dissociation constant of a negatively charged amino acid or the C-terminal and *pK*_*p *_is the acid dissociation constant of a positively charged amino acid or the N-terminal. The net charge of the protein equals to the sum of *y*_*n *_and *y*_*p*_. Therefore, by experimenting with different pH values, we can locate the one that results in the charge sum to be zero. This pH value is then the isoelectric point of the protein under investigation. In our implementation, Nozaki and Tanford's pK table [9] has been used.

To take the factor of hydrophobicity index profile into consideration, we initiate a statistical analysis on the sequences from the Phase II-Intermediate data set to facilitate the selection of the sequences whose HI fit in the average HI profile for the expressed proteins in this organism. The procedure is explained as follows:

1. For each sequence in Phase II-Intermediate data collection, obtain its hydropathy plot using the Kyte-Doolittle scale [10] and record the values for the following five variables (where positive includes 0 in this case):

(a) the maximal hydrophobicity index

(b) the minimal hydrophobicity index

(c) the average positive hydrophobicity index

(d) the average negative hydrophobicity index

(e) the percentage of residues having positive hydrophobicity index

2. For each of the five metrics mentioned above, calculate the mean  and the sample variance *S *using Eq. 4 and Eq. 5 respectively.

(4)

(5)

3. Filter the sequences in the Phase II-Intermediate collection so that a qualifiable sequence has a hydrophobicity profile whose values for the above 5 variables lie within the range of  for any individual variable. It is worth noting that according to the empirical rule [11], in the case of a normal distribution, 95% of the population will lie within this range.

In Phase III, we further eliminate some sequences that are not suitable for TIS predictor evaluation from Phase II if one meets at least one of the following two conditions: *a*) the number of the nucleotides in the 5' *untranslated region *(UTR) is less than 12, and *b*) if the protein that corresponds to the mRNA sequence belongs to a homologous protein family and an mRNA that represents this group of proteins has already been included in the data set.

The reason for having condition *a *is to facilitate the usage and increase the efficiency of the data set for TIS prediction because if untrimmed, the transcript sequence collection, when tested on most of the established TIS classifiers, may require considerably more computational resources than that is normally able to be offered. Since most existing systems consider a neighborhood window that is upstream to a putative start codon, which usually spans from positions -12 to -1 [12,13], we only consider the sequences that have a 5' UTR which at least contains 12 nucleotides. We realize that such a strategy may not be valid for testing any approach which relies on a 5' UTR of a wider range. Should such a demand becomes a reality, it is trivial to reconstruct the data sets by following the generalized post-processing algorithm that is presented in the ensuing text. We denote the data set that include only the sequences having 5' UTR of 12 or more nucleotides by the Phase III-Intermediate collection.

Having a check on condition *b *avoids selecting redundant transcript sequences that lead to homologous proteins. We used the BLAST tool [14] to analyze homology between a pair of sequences. The procedure is described as follows:

1. Construct a BLAST database using only the protein sequences that correspond to the entries in the Phase III-Intermediate collection.

2. Initialize two empty entry ID lists for the qualifiable and the redundant sequences, which are denoted by *L*_*q *_and *L*_*r *_respectively.

3. For each sequence in the collection under investigation, check whether its ID is in *L*_*r*_. If so, skip this sequence and continue with the next one in the data set. Otherwise, use *blastp *to locate the entries that are homologous to the query entry (with a threshold value for *e *of 1e-30). Add those entry IDs to *L*_*r*_.

4. Repeat Step 3 until every sequence has been scanned.

To complete the construction of the Phase III sequence collection, we have employed a post-processing procedure. Although a complete mRNA sequence contains the 5' cap, the 5' UTR, the 3' UTR and a poly-A tail in addition to the CDS, not all of these regions are of critical importance to identify the initiation site for translation. Therefore we conduct a randomized trimming process on the sequences in the collection. The details of the post-processing strategy follows:

1. Set **max**_*u *_and **min**_*u *_to be the maximal and the minimal numbers of nucleotides in the 5' UTR after trimming. Set **max**_*d *_and **min**_*d *_to be the maximum and the minimum of the size of the 3' UTR after trimming.

2. For 5' UTR:

(a) obtain a random number **rand**_*u *_ranging from **min**_*u *_to **max**_*u*_

(b) find the lesser value of **rand**_*u *_and **start_codon_position**, denote it by **margin**_*u*_

(c) trim the 5'UTR region so that the rightmost **margin**_*u *_nucleotides are remained.

3. Keep the entire CDS.

4. For 3' UTR:

(a) obtain a random number **rand**_*d *_ranging from **min**_*d *_to **max**_*d*_

(b) find the lesser value of **rand**_*d *_and (**sequence_length – stop_codon_position**), denote it by **margin**_*d*_

(c) trim the 3' UTR region so that the leftmost **margin**_*d *_nucleotides are remained.

In our implementation, **max**_*u *_= 40, **min**_*u *_= 12, **max**_*d *_= 40, and **min**_*d *_= 0.

## Results and Discussion

### Data Sets

We have applied the aforementioned algorithms to generate four representative transcript sequence collections for the following model organisms: *Homo sapiens *(human), *Mus musculus *(house mouse), *Caenorhabditis elegans *(roundworm) and *Drosophila melanogaster *(fruit fly). A quick summary of the number of sequences in the data sets at different phases is shown in Table 1.

**Table 1:** Statistics regarding the cardinality and size portion of the data sets

	Phase I		Phase II		Phase III	
	
	Card.	Ratio	Card.	Ratio	Card.	Ratio
*H. sap*.	25697	100%	8006	31%	2498	10%

*M. mus*.	21031	100%	6723	32%	2504	12%

*C. ele*.	23758	100%	7883	33%	1342	7%

*D. mel*.	20096	100%	5900	29%	1670	8%

The cardinality of a data set at a particular phase (denoted by Card.) and the size portion of the Phase I collection that has been retained in this phase (denoted by Ratio).

As we desired to obtain data sets of transcripts whose expressed polypeptide products were representative of proteins most commonly expressed in cells, comparison with the protein universe identified in proteomic studies was in order. Unfortunately, published studies showing the physical distribution of proteins identified by proteomic analysis are relatively uncommon, far outnumbered by those evaluating specific protein classes and/or concerned with protein or protein subdomain identification and classification [15,16]. A very thorough proteomic analysis of 8 different human tissues did show distributions of proteins within protein structural parameters such as molecular weight, pI and hydrophobicity that we could use for comparison [17]. From their data, approximately 80% of expressed proteins distribute between 10–75 KDa MW. Approximately 78% of proteins segregate between pI 5–9. As well, a very good normal distribution of protein hydrophobicity was observed, with approximately 94% of proteins distributing within the middle ranges (GRAVY score 20–70) of hydrophobicity. In a study of the mouse liver proteome using advanced mass spectrometry [18], similar findings were also revealed. From the 3D plot showing the UniProt complete mouse proteome, a majority of the expressed proteins lie in the range of 10–100 KDa MW and 5–9 for pI. Although it is premature to assume that these ranges would apply to any organism, they have however reflected the actual mammalian proteomic data. Having a focus of presenting a general algorithm for selecting the transcript sequences that yieid a set of proteins satisfying particular parameter settings, we decide to adopt a sample scheme for configuring the MW and pI parameters based upon our experience with the proteomic literature concerning human proteins where MW is within the range of 20–70 KDa and pI is between 5–9. It is worth noting that the filtration of the data sets in Phase II is arbitrary and it is not intended to serve as a universal strategy for every organism.

**Figure 1:**
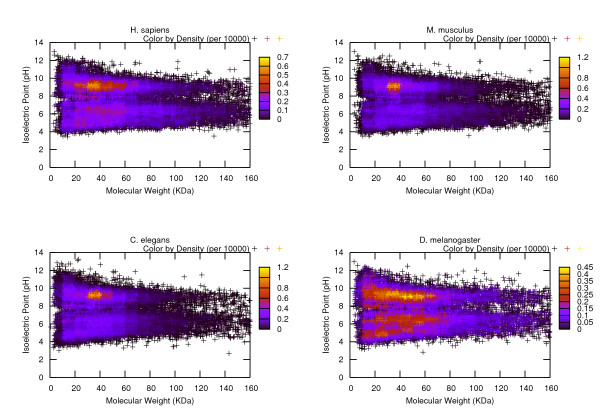
**Presentation of the MW-pI plot for Phase I data sets**. Each sequence entry's corresponding protein is represented by a (MW, pI) tuple, therefore, the entire collection can be plotted onto a two-dimensional space. To highlight the difference of population densities among different regions of the graph, a variable called density is employed. It controls the color of the scattered points on the plot, where the plot space is viewed as a grid that consists of many small cells each of which has a length of 10 KDa along the X-axis and a width of 0.1 pH along the Y-axis. Each cell's local population (the number of points that fall into that region) is recorded and its ratio over the entire population is calculated. Since most of the ratios are very small numbers, they have been multiplied by 10000 before they are used in plotting the graph.

**Figure 2:**
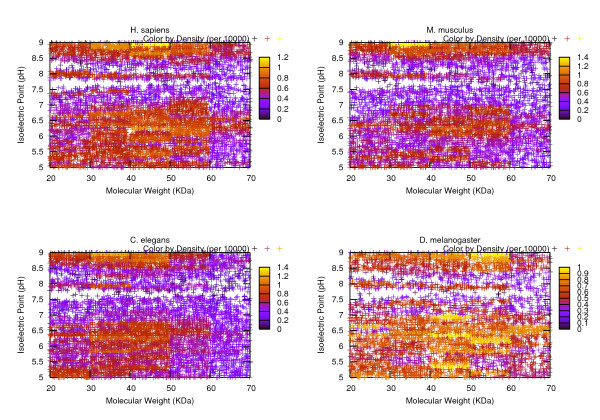
**Illustrates the MW-pI plot for Phase II data sets**. Compared to Figure 1, narrower value ranges are used for MW and pI, where MW is in between 20–70 KDa, and pI is in between 5 to 9 pH.

**Figure 3:**
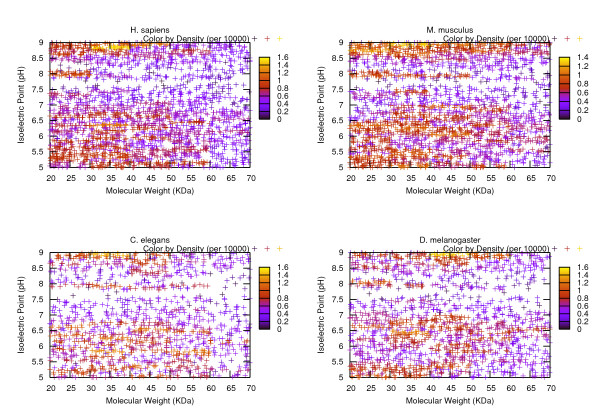
**A visual presentation of the Phase III data sets' MW-pI plot**. In obtaining the data sets at this phase, more restrictions are applied, making the distributions of scattered points even more sparse than Figure 2.

In order to provide a reference of the characteristics of the average protein groups in each of these organisms, we conducted some statistical analysis on them based on the measurements of MW, pI and the hydropathy plots. Figures. 1, 2, 3 visualize the transcripts' c orresponding proteins' MW vs pI plots for each of the three phases. Table 2 reports the statistics related to a variety of MW ranges given the data sets of three phases. Table 3 summarizes the results of the statistical analysis conducted from the perspective of isoelectric point. All of the remaining tables are dedicated to the protein hydropathy analysis. Since for a given protein, a continuous hydropathy plot can be obtained, we try to capture its characteristics by analyzing the following aspects. Tables 4, 5 refer to the analysis conducted on the maximal and minimal hydrophobicity values within the hydrophobicity plot. Tables 6, 7 illustrate the results regarding the average positive hydrophobicity values and the average negative hydrophobicity values. Table 8 reports a series of statistics that are relevant to the percentage of hydrophobic residues within the plot.

**Table 2:** Statistics of the proteins' molecular weight

		[0, 20)		[20, 40)		[40, 60)		[60, 80)		[80, 100)		[100, 120)		[120, 140)		[140, +∞)	
		
		PR	LR	PR	LR	PR	LR	PR	LR	PR	LR	PR	LR	PR	LR	PR	LR
*H. sap*.	I	13%	0%	***28%***	0%	***23%***	0%	***13%***	0%	8%	0%	5%	0%	3%	0%	7%	0%
	
	II	0%	100%	***40%***	55%	***45%***	39%	15%	64%	0%	100%	0%	100%	0%	100%	0%	100%
	
	III	0%	100%	***47%***	83%	***39%***	83%	14%	89%	0%	100%	0%	100%	0%	100%	0%	100%

*M. mus*.	I	11%	0%	***32%***	0%	***23%***	0%	***13%***	0%	8%	0%	5%	0%	3%	0%	6%	0%
	
	II	0%	100%	***41%***	59%	***44%***	38%	15%	63%	0%	100%	0%	100%	0%	100%	0%	100%
	
	III	0%	100%	***46%***	82%	***40%***	79%	14%	87%	0%	100%	0%	100%	0%	100%	0%	100%

*C. ele*.	I	19%	0%	***33%***	0%	***24%***	0%	***11%***	0%	5%	0%	3%	0%	2%	0%	3%	0%
	
	II	0%	100%	***43%***	56%	***44%***	39%	13%	60%	0%	100%	0%	100%	0%	100%	0%	100%
	
	III	0%	100%	***44%***	92%	***44%***	89%	13%	93%	0%	100%	0%	100%	0%	100%	0%	100%

*D. mel*.	I	14%	0%	***25%***	0%	***22%***	0%	***14%***	0%	8%	0%	5%	0%	3%	0%	8%	0%
	
	II	0%	100%	***38%***	55%	***45%***	39%	17%	64%	0%	100%	0%	100%	0%	100%	0%	100%
	
	III	0%	100%	***44%***	85%	***41%***	84%	14%	91%	0%	100%	0%	100%	0%	100%	0%	100%

This table reports the statistics of the molecular weight of the proteins corresponding to the transcript sequences included in Phases I, II, III data sets (Unit: KDa). To facilitate the analysis, the entire MW value range has been divided into several smaller ranges (e.g., 0–20 KDa, 20–40 KDa, etc). Throughout the table, two measures have been employed: PR, short for Population Ratio, is a horizontal comparison which compares the protein population within a group against other groups in the same phase; whereas LR, short for Loss Ratio, is a vertical comparison which, for a certain group, considers the portion of the protein population that have been lost through phase transitions. More specifically, given a particular aspect X under investigation (e.g. MW, pI, etc), PR calculates the portion of the number of proteins whose value on X is within a particular range (e.g, [20, 40) KDa, [8, 9) pH, etc) over that of the entire protein population in the same phase. LR computes the ratio of the number of eliminated proteins whose value on X is within a particular range Y over that of the original (Phase I) protein population within that range Y. The data that have been highlighted in italic and bold font are the outstanding numbers within the row it belongs to.

**Table 3:** Analysis of the data sets at Phases I, II, and III by isoelectric point (Unit: pH)

		[0, 5)		[5, 6)		[6, 8)		[8, 9)		[9, 14]	
		
		PR	LR	PR	LR	PR	LR	PR	LR	PR	LR
*H. sap*.	I	8%	0%	***18%***	0%	***31%***	0%	***16%***	0%	26%	0%
	
	II	0%	100%	27%	53%	***47%***	52%	25%	51%	1%	98%
	
	III	0%	100%	30%	83%	***44%***	86%	25%	84%	1%	99%

*M. mus*.	I	8%	0%	***17%***	0%	***30%***	0%	***18%***	0%	27%	0%
	
	II	0%	100%	26%	51%	***46%***	50%	28%	50%	1%	98%
	
	III	0%	100%	28%	80%	***44%***	82%	27%	82%	1%	99%

*C. ele*.	I	10%	0%	***17%***	0%	***28%***	0%	***17%***	0%	28%	0%
	
	II	0%	100%	28%	45%	***45%***	46%	26%	49%	1%	98%
	
	III	0%	100%	30%	90%	***46%***	90%	24%	92%	0%	100%

*D. mel*.	I	9%	0%	***17%***	0%	***32%***	0%	***16%***	0%	27%	0%
	
	II	0%	100%	27%	53%	***48%***	55%	24%	55%	1%	98%
	
	III	0%	100%	28%	86%	***46%***	88%	25%	87%	1%	99%

**Table 4:** Statistics regarding the hydropathy plot

		[0, 1)		[1, 2)		[2, 3)		[3, 4)		4+	
		
		PR	LR	PR	LR	PR	LR	PR	LR	PR	LR
*H. sap*.	I	1%	0%	11%	0%	***49%***	0%	***36%***	0%	2%	0%
	
	II	0%	100%	7%	80%	***56%***	63%	34%	70%	0%	100%
	
	III	0%	100%	8%	92%	***59%***	88%	33%	91%	0%	100%

*M. mus*.	I	1%	0%	10%	0%	***46%***	0%	***40%***	0%	3%	0%
	
	II	0%	100%	6%	80%	***55%***	61%	37%	70%	2%	78%
	
	III	0%	100%	8%	90%	***56%***	85%	34%	89%	1%	96%

*C. ele*.	I	1%	0%	11%	0%	***43%***	0%	***42%***	0%	3%	0%
	
	II	0%	100%	7%	78%	***54%***	58%	38%	69%	1%	88%
	
	III	0%	100%	8%	95%	***55%***	92%	36%	95%	1%	98%

*D. mel*.	I	1%	0%	11%	0%	***49%***	0%	***36%***	0%	3%	0%
	
	II	0%	100%	7%	81%	***59%***	64%	34%	72%	0%	100%
	
	III	0%	100%	9%	93%	***61%***	89%	31%	92%	0%	100%

This table reports the statistics regarding the analysis conducted on the corresponding protiens' hydropathy plot using Kyte-Doolittle scale. The particular parameter that is investigated is called the maximal hydrophobicity, which corresponds to the global maximal value of a continuous hydropathy plot.

**Table 5:** Statistics regarding the analysis of the corresponding proteins' hydropathy plot

		Below -4		[-4, -3)		[-3, -2)		[-2, -1)		[-1, 0)	
		
		PR	LR	PR	LR	PR	LR	PR	LR	PR	LR
*H. sap*.	I	5%	0%	***69%***	0%	***25%***	0%	1%	0%	0%	0%
	
	II	1%	93%	***70%***	68%	28%	65%	0%	100%	0%	0%
	
	III	1%	98%	***68%***	90%	32%	87%	0%	100%	0%	0%

*M. mus*.	I	4%	0%	***66%***	0%	***28%***	0%	1%	0%	0%	0%
	
	II	1%	92%	***67%***	67%	33%	62%	0%	100%	0%	0%
	
	III	1%	97%	***66%***	88%	33%	85%	0%	100%	0%	0%

*C. ele*.	I	3%	0%	***62%***	0%	***32%***	0%	2%	0%	0%	0%
	
	II	1%	88%	***67%***	64%	32%	66%	0%	100%	0%	0%
	
	III	1%	98%	***69%***	93%	30%	94%	0%	100%	0%	0%

*D. mel*.	I	6%	0%	***69%***	0%	***24%***	0%	1%	0%	0%	0%
	
	II	1%	95%	***70%***	70%	28%	65%	0%	100%	0%	0%
	
	III	1%	98%	***70%***	91%	28%	90%	0%	100%	0%	0%

This table focuses on the analysis of minimal hydrophobicity, which corresponds to the global minimal value of a continuous hydrophobicity plot.

**Table 6:** Statistics of proteins' hydropathy plots at different phases

		[0, 1)		[1, 2)		[2, 3)		[3, 4)		4+	
		
		PR	LR	PR	LR	PR	LR	PR	LR	PR	LR
	
*H. sap*.	I	***81%***	0%	19%	0%	0%	0%	0%	0%	0%	0%
	
	II	***87%***	66%	13%	78%	0%	0%	0%	0%	0%	0%
	
	III	***86%***	89%	14%	92%	0%	0%	0%	0%	0%	0%
*M. mus*.	I	***76%***	0%	24%	0%	0%	0%	0%	0%	0%	0%
	
	II	***82%***	65%	18%	76%	0%	0%	0%	0%	0%	0%
	
	III	***83%***	86%	17%	91%	0%	0%	0%	0%	0%	0%

*C. ele*.	I	***72%***	0%	28%	0%	0%	0%	0%	0%	0%	0%
	
	II	***84%***	61%	16%	81%	0%	0%	0%	0%	0%	0%
	
	III	***85%***	93%	15%	96%	0%	0%	0%	0%	0%	0%

*D. mel*.	I	***81%***	0%	18%	0%	0%	0%	0%	0%	0%	0%
	
	II	***89%***	67%	11%	82%	0%	0%	0%	0%	0%	0%
	
	III	***89%***	90%	11%	94%	0%	0%	0%	0%	0%	0%

The table reports the statistics of the data sets at different phases with the focus on the proteins' hydropathy plot. The particular metric called average positive hydrophobicity is computed by averaging all the hydrophobicity index values that are higher than or equal to 0 within one plot.

**Table 7:** A summary of the data on average negative hydrophobicity

		Below -4		[-4, -3)		[-3, -2)		[-2, -1)		[-1, 0)	
		
		PR	LR	PR	LR	PR	LR	PR	LR	PR	LR
*H. sap*.	I	0%	0%	0%	0%	0%	0%	***65%***	0%	35%	0%
	
	II	0%	0%	0%	0%	0%	0%	***56%***	72%	42%	62%
	
	III	0%	0%	0%	0%	0%	0%	***56%***	91%	42%	88%

*M. mus*.	I	0%	0%	0%	0%	0%	0%	***63%***	0%	37%	0%
	
	II	0%	0%	0%	0%	0%	0%	***56%***	71%	43%	62%
	
	III	0%	0%	0%	0%	0%	0%	***56%***	89%	43%	86%

*C. ele*.	I	0%	0%	0%	0%	0%	0%	***62%***	0%	38%	0%
	
	II	0%	0%	0%	0%	0%	0%	***56%***	70%	43%	62%
	
	III	0%	0%	0%	0%	0%	0%	***61%***	94%	39%	94%

*D. mel*.	I	0%	0%	0%	0%	0%	0%	***69%***	0%	31%	0%
	
	II	0%	0%	0%	0%	0%	0%	***59%***	74%	41%	61%
	
	III	0%	0%	0%	0%	0%	0%	***62%***	92%	38%	89%

This parameter is yielded by averaging over all the hydrophobicity values that are less than 0 within one plot.

**Table 8:** Statistics of hydrophobic residues within the context of hydropathy plots

		[0, 20)		[20, 40)		[40, 60)		[60, 80)		[80, 100]	
		
		PR	LR	PR	LR	PR	LR	PR	LR	PR	LR
*H. sap*.	I	7%	0%	***54%***	0%	***33%***	0%	6%	0%	0%	0%
	
	II	2%	91%	***56%***	67%	***41%***	61%	1%	94%	0%	0%
	
	III	3%	95%	***53%***	90%	***43%***	87%	2%	96%	0%	0%

*M. mus*.	I	6%	0%	***50%***	0%	***33%***	0%	10%	0%	0%	0%
	
	II	2%	89%	***51%***	67%	***42%***	59%	5%	84%	0%	0%
	
	III	3%	94%	***52%***	87%	***41%***	85%	4%	95%	0%	0%

*C. ele*.	I	7%	0%	***50%***	0%	***33%***	0%	10%	0%	0%	0%
	
	II	2%	90%	***55%***	63%	***40%***	59%	2%	93%	0%	0%
	
	III	3%	97%	***56%***	93%	***36%***	93%	3%	98%	0%	0%

*D. mel*.	I	7%	0%	***56%***	0%	***32%***	0%	4%	0%	0%	0%
	
	II	2%	91%	***56%***	70%	***40%***	63%	1%	92%	0%	0%
	
	III	3%	96%	***60%***	91%	***37%***	90%	1%	97%	0%	0%

Given one continuous plot, the percentage is calculated by dividing the number of nucleotide residues that correspond to a positive (0 included) hydropathy index by that in a complete ORF.

Some observations can be made from these illustrations. Interestingly, the significant density differences observed within the MW-pI plots (Figures. 1, 2, 3) show the non-random nature of the protein expression spectrum within the "average" mRNA set. For instance, from the Phase I plot, we can see that for all of the four model organisms, the region with the biggest density (shown in yellow hue) is within the range of [20K, 70K] Da for MW and [5,9] pH for pI. This verifies the hypothesis we made earlier about the average profile of proteins in terms of MW and pI. As well, in a vertical comparison among the population, as the value of molecular weight gets bigger, the value range of the corresponding pI becomes narrower, demonstrating a tendency of converging to the [5,9] range as MW increases. Since a majority of the sequences have been eliminated from the Phase I data set due to the subsequent selection procedure, Figures 2, 3 are much more sparse than Figure 1. It is especially easy to n otice two horizontal gaps in both plots where the pI value is in between [7.5, 8) and (8, 8.5] pH. This is an indicator of the sparse distribution of the protein population whose pI lies within those two ranges. A table that provides the counts of the proteins fall under those two categories would suffice to verify our interpretation of the visual data (see Table 9). From the table we can see that in either Phase II or Phase III, since only 3 to 8 percent of the protein population have a pI value falling under those two ranges, the density in those horizontal areas is fairly small, thus the gap is shown. In order to verify our hypothesis that the gap is a result of some statistically significant reason other than a coincidence, we have conducted a paired statistical t-test. The procedure is summarized as follows. Assume the population of the organism are distributed at a random manner within the pI range 5 to 9. Therefore, for a horizontal stripe whose pI value ranges from 7.5 to 7.9 or 8.1 to 8.5, roughly 1/10 of the entire population should fall into that stripe. If we consider both regions ([7.5, 7.9] and [8.1, 8.5]), we should expect 2/10 of the population. Using the four model organisms' related data as the sample data, a two-tailed t-test with type I error of 0.05 has a *p *value of 0.0007 for Phase II data set and a *p *value of 0.0059 for Phase III data set. Therefore, either case should be considered as extremely statistically significant, indicating that the sparse distribution in those two regions is not a result of randomness.

**Table 9:** The population density of the proteins having a pI value within range [7.5, 7.9] or [8.1, 8.5]

		*N*_0_	*N*_1_	*R*_1_	*N*_2_	*R*_2_
*H. sap*.	II	8006	365	4.56%	548	6.84%
	
	III	2498	95	3.80%	163	6.53%

*M. mus*.	II	6723	306	4.55%	507	7.54%
	
	III	2504	95	3.79%	188	7.51%

*C. ele*.	II	7883	337	4.28%	577	7.32%
	
	III	1342	57	4.25%	93	6.93%

*D. mel*.	II	5900	246	4.17%	401	6.80%
	
	III	1670	51	3.05%	103	6.17%

This table provides an illustration of the population density of the proteins that have a pI value within range [7.5, 7.9] or [8.1, 8.5] for Phases II, III data sets. *N*_0 _denotes the total count of the proteins in Phase II or Phase III data set; *N*_1 _refers to the number of proteins whose pI is in [7.5, 7.9], *R*_1 _is equal to *N*_1 _over *N*_0_; and *N*_2 _denotes the population of the proteins having an isoelectric point in range [8.1, 8.5] and *R*_2 _is its corresponding ratio.

Interestingly, the bimodal distribution observed by us and also by proteomic analysis of human and mouse proteins [17,18] is also observed in studies evaluating the pI distribution of several expressed genomes [19,20], using completely different methodology than that used to generate our 4 transcript data sets. This distribution of pI values is alleged to have functional significance in correlating with subcellular protein distributions [19]. Another rationale we would provide for this observation could be a biological selection to avoid proteins whose pI values fall near functional cellular pH values. Such proteins might be prone to aggregation or precipitation at those pH values. Currently, several databases exist that could allow comparison of structural characteristics of expressed proteins between organisms, however, such additional comparisons are beyond the aims of our current study [15,16,21-23].

Tables 2, 3 summarize further analysis conducted on the protein populations in different phases on MW and pI values. From the entries regarding Phase I collection, we can see that more than 75% of the population fall under the MW range of [0, 80K] Da with the majority lie within the range of [20K, 40K)Da. As well, more than 90% of the proteins satisfy the pI condition of ranging between 5 and 14 pH, with the majority fall under the range of 6–8. The data related to the Phase II and Phase III data sets reflect that consistently about 45% of the proteins have an isoelectric point that is in between 6 and 8 pH, making us believe in these four model organisms, a majority of the proteins that fall under an average profile are neutral.

Tables 4 to 8 have shown the hydrophobicity profile analysis (using Kyte-Doolittle scale) conducted on the data sets from different phases. In Phase I, generally speaking, more than 85% of the proteins have a hydrophobicity profile whose peak value is in between 2 and 4, more than 62% of the proteins have a HI profile with a lowest value that is in between -4 and -3, the average values for positive and negative hydropathy lie in the ranges of [0, 1] and [-2, -1] respectively, and the average percentage of hydrophobic residues falls under the range of [20%, 60%] for a majority of the protein population. Due to the selection process, collections in subsequent phases demonstrate that value ranges on the aforementioned five aspects have been narrowed. For instance, now in most of the cases, the maximal hydropathy value is 2 to 3 and the minimal one is -4 to -3. Although these information are still quite limited for providing a comprehensive representation of the hydrophobicity profiles associated with all of the proteins under investigation, they do offer some means of interpreting the hydropathy index plots.

**Figure 4:**
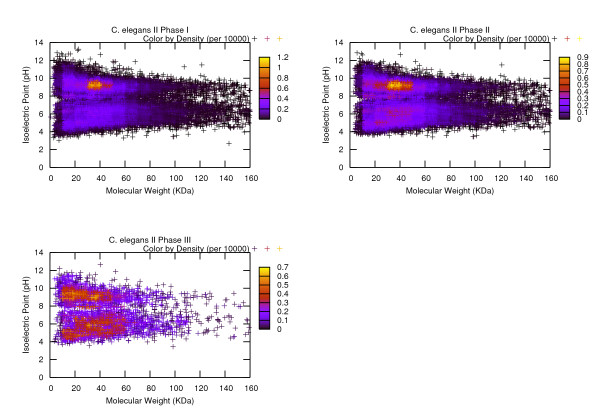
**Presentation of the MW-pI plots for the C. elegans II data sets in three phases**. These plots represent the distribution of the Phase I, II, III data sets for *C. elegans II *for the purpose of demonstrating the applicability of the proposed data set generation method in the absence of *a priori *parameter configuration scheme. We suggest the user to divide the entire ranges of the parameters (e.g. MW and pI) into subgroups and use the proposed algorithm to handle each subgroup individually. Ultimately a representative data set is obtained by combining all of the subgroups together. From the plots we can observe that eventually (in Phase III) the most populated regions converge to some certain ranges, i.e., MW in between 20–60 KDa and pI in between 4 to 10. A bimodal pattern can also be observed.

It is important to note that so far for all of the four organisms, we have used a general range configuration for both MW and pI, which are 20–70 KDa and 5–9 respectively. Although this setting tends to reflect the characteristics of mammalian proteomic data (e.g. *H. sapiens *and *M. musculus*), it is more or less an arbitrary choice for *C. elegans *and *D. melanogaster*. However, the benchmark generation procedure that we present in this paper is independent of the values of the parameters that are predefined by the user. To tailor towards the characteristics of the expressed proteins in a particular species, a thorough investigation of the molecular weight and isoelectric point of the proteins may be necessary. If such investigative operation is not desired, the user may choose to follow a strategy described as follows: the complete valid ranges for MW and pI can be divided up into several groups according to the convention observed in the proteomic study (Table 2 and Table 3 offer an example of this tactic), then each of these sub-ranges are used individually to specify the parameter setting for the benchmark generation procedure and in the end, all of the resulting data sets are put together to comprise the representative sequence collection for the given organism. To demonstrate the generality and applicability of the proposed approach, we have used the aforementioned strategy to construct a second set of transcript collection for *C. elegans *and we refer to it as the *C. elegans II*. During the production process, we have used rather extensive ranges for the parameters MW (0–160 KDa) and pI (0–14) and divided them up into smaller sub-ranges which are consistent with the ones seen from Table 2 and Table 3. In the end, we combine all of the subgroups together to arrive at the complete representative sequence collections. Figure 4 illustrates the MW-pI plots for each of the three phases that the data set construction process has undergone. From the plots we can observe that eventually (in Phase III) the most populated regions converge to certain ranges, i.e., MW in between 20–60 KDa and pI in between 4 to 10. A bimodal pattern can also be observed. The applicability of our strategy is therefore well demonstrated.

### Evaluation

In order to demonstrate the applicability of the proposed algorithm, we have employed several state-of-the-art predictors for further evaluation, including NetStart [1], CUB [24], TISHunter [25], StartScan [4], GENSCAN [26] and GlimmerHMM [27], where the first four software are specialized TIS predictors and the latter two are designed to predict entire genes. Nevertheless, both GENSCAN and GlimmerHMM can be indirectly used to identify translational initiation sites. With the exception of NetStart, which may predict multiple start codons within one gene, all of the other approaches assume that one and only one TIS exists per gene. Other than CUB, which is proposed by us, the other five predictors all require a training process and the pre-trained models are made available to the academic users free of charge. Therefore the experimental results can be obtained by directly using the transcript sequence collections we constructed as the test set. All of these peer predictors have been pre-trained for certain model organism(s) using some data sets that have not been revealed to the user. For instance, NetStart is trained for handling vertebrates and *Arabidopsis thaliana *sequences, TISHunter can predict TIS for both *H. sapiens *and *M. musculus*, StartScan is capable of recognizing TIS in *H. sapiens *genomic sequences, GENSCAN has been trained for vertebrate, *Arabidopsis thaliana *and maize, and GlimmerHMM is able to analyze sequences from the genomes of *H. sapiens, Arabidopsis thaliana *and rice. As far as CUB is concerned, we applied the transcript collections as the testing sets directly and collected the experimental results in that the model does not need to be trained. To summarize, we used the NetStart model pre-trained on vertebrates to predict TIS in the data sets of *H. sapiens *and *M. musculus*. The same two data sets are tested by TISHunter using the model pre-trained on human sequences (the author declares that it is able to predict TIS in mouse sequence well too).

We have employed four evaluation metrics to measure the performance of the TIS predictor: *sensitivity *(Sn), *specificity *(Sp), *Adjusted Accuracy *(AA) and *Overall Accuracy *(OA). A high sensitivity indicates a big portion of true TISs have been predicted. A high specificity means a big portion of pseudo-TISs have been accurately recognized by the predictor. Due to the evaluation bias each of these two metrics imposes, it is prudent not to discuss them in isolation. Instead, adjusted accuracy, which is the average of the two, serves as a more comprehensive metric. Each of these measures can be easily computed if the contingency matrix shown in Table 10 is available. The formulas are listed as follows:

**Table 10:** Contingency Matrix

	Classified as True	Classified as False
Actual True	TP	FN

Actual False	FP	TN



Table 11 reports all of the results that are available using the transcript data collections we constructed. From the data we can see that all of the systems have demonstrated promising performance on our data sets with GENSCAN, TISHunter and CUB being the front-runners.

**Table 11:** Experimental results using six existing TIS predictors on our transcript collections

Data Set	Approach	Sn	Sp	AA	OA
*H. sap*.	NetStart	83.59%	69.35%	76.47%	70.00%
	
	TISHunter	94.99%	99.79%	97.39%	99.57%
	
	CUB	80.14%	99.04%	89.54%	98.17%
	
	StartScan	96.72%	61.29%	79.01%	62.92%
	
	GENSCAN	79.02%	99.11%	89.07%	98.19%
	
	GlimmerHMM	68.29%	98.39%	83.34%	96.94%

*M. mus*.	NetStart	83.43%	68.00%	75.71%	68.70%
	
	TISHunter	70.42%	98.76%	84.59%	97.48%
	
	CUB	79.11%	99.01%	89.06%	98.11%
	
	GENSCAN	84.07%	99.35%	91.71%	98.66%

*C. ele*.	CUB	81.30%	99.28%	90.29%	98.62%

*D. mel*.	CUB	83.65%	99.19%	91.42%	98.45%

*C. ele. II*	CUB	79.99%	99.19%	89.59%	98.45%

Four metrics are employed in our experiments: sensitivity (*Sn*), specificity (*Sp*), adjusted accuracy (*AA*) and overall accuracy (*OA*).

To provide a more complete comparative study, we present Table 12[24] which includes the results of using NetStart, CUB and GENSCAN on three other benchmark data sets that we discussed earlier in this paper – vertebrates, *Arabidopsis thaliana *and *TIS+50*. To test NetStart on the data sets vertebrates, *Arabidopsis thaliana *and *TIS+50*, the organisms that the applied model are trained for are vertebrates, *Arabidopsis thaliana *and vertebrates respectively. As far as GENSCAN is concerned, the corresponding organisms are vertebrates, *Arabidopsis thaliana *and vertebrates. All of the models that are used in the testing are trained and provided by the original authors of the software.

**Table 12:** Experimental results using three existing TIS predictors on benchmark collections

Data Set	Approach	Sn	Sp	AA	OA
*vert*	NetStart	82.25%	87.80%	85.02%	86.44%
	
	CUB	89.58%	96.61%	93.10%	94.89%
	
	GENSCAN	0.24%	90.25%	45.24%	68.17%

*Arab*	NetStart	97.32%	88.79%	93.06%	90.97%
	
	CUB	91.78%	97.18%	94.48%	95.80%
	
	GENSCAN	0.57%	89.31%	44.94%	66.65%

*TIS+50*	NetStart	88.00%	69.93%	78.97%	71.78%
	
	CUB	80.00%	97.72%	88.86%	95.91%
	
	GENSCAN	64.00%	98.41%	81.20%	94.89%

This table reports the results using three TIS predictors on vertebrates (*vert*.), *Arabidopsis thaliana *(*Arab*.) and *TIS+50 *data sets.

A brief comparison between the data shown in Table 11 and Table 12 identifies the following: (1) GENSCAN, as a leading gene predictor, fails to perform adequately on the vertebrates and *Arabidopsis thaliana *collections while its performance on our *H. sapiens *data set seems more reasonable. This may be an indication of the extra trimming procedure conducted on the sequences included in the former two collections (or NetStart). Our explanation is described as follows. First of all, GENSCAN is mainly dedicated to predicting genes in genomic sequences. Therefore for any predicted gene, at most one AUG will be classified as TIS. The sequences in the NetStart collections have undergone extensive trimming process, resulting in the average length of these sequences to be only 150 nucleotides long. Therefore, if the GENSCAN software fails to identify a sequence in NetStart as the start of a gene, it will not make a positive TIS prediction, thus yielding a small true positive and a large false negative, which implies a small sensitivity. Secondly, GENSCAN relies heavily on the statistics obtained by analyzing long stretch of genomic sequences and it also takes advantage of compositional bias between exons and introns. However the NetStart sequences are simply too short and none of them contain an entire open reading frame, which consequently lead to the surprisingly low performance offered by GENSCAN; (2) other than CUB, which demonstrates consistent performance on all of the data sets shown in Table 11 and Table 12, most of the peer predictors including NetStart, GENSCAN and TISHunter have shown inconsistent prediction performance on different genomes.

## Conclusion

In this paper, we have presented a general procedure for constructing representative mRNA sequence collections for the purpose of testing translational initiation site recognition approaches. We believe an ideal benchmark test set should contain high quality mRNA sequences that are error-free and contain complete ORFs. It should also be highly representative, non-redundant and easily accessible. One definition for a "representative" data set would be for the expressed proteins to be functionally representative of what is obtained from proteomic analysis. Thus, the expressed protein data set should approximate the published distribution of proteins from proteomic analysis.

To the best of our knowledge, very few data sets have been widely used for evaluating an existing TIS prediction approach. Most of the collections proposed in the literature violate one or more of the aforementioned rules. To ensure the reliability of our data sets, we only selected the mRNA sequences from the NCBI RefSeq database, which is a high quality, non-redundant sequence repository that has been curated either manually or by specialized programs. We analyze the characteristics associated with each mRNA's corresponding protein product and conduct further elimination on the data set to remove the sequences that do not correspond to proteins which present an average profile for a given organism. In order to avoid the possibility of exaggerating the performance of an approach to be tested, for a family of homologous proteins, only one corresponding mRNA sequence is retained to represent the group of homologous sequences. It is worth noting that though redundancy elimination through homology examination is a common routine for benchmark constructions, the homology in proteins does not necessarily indicate similar context around TIS, which may distort the accuracy of a predictor. However, with the intention of proposing a general strategy for benchmark collection generation, we still choose to use homology as a guideline to eliminate redundant copies of homologous proteins, hoping that the resulting collections can be used to more general areas such as proteomic investigation.

Four model organisms have been used as an example to illustrate the construction procedure and to demonstrate its property of being a general and widely applicable scheme. Every data set in every phase has been analyzed in terms of the molecular weight, isoelectric point and hydrophobicity profile of the corresponding protein products. Colored plots have also been used to help visualize the data yielded by our analysis. Our comparison with published proteomic studies has shown that expression of our data set of transcripts generates a polypeptide population that is representative of that obtained from evaluation of biological specimens. Hence, we believe that our data set represents "real world" transcripts that will allow more accurate evaluation of algorithms dedicated to identification of TISs, as well as other translational regulatory motifs within mRNA sequences. Although during the process of constructing these four data sets, we have selected a specific range for the parameters (MW and pI), we have also proposed a strategy that can be coupled with the proposed algorithm when the user does not intend to impose any pre-defined parameter setting. A sample collection on *C. elegans *has been constructed to demonstrate the applicability of the proposal.

Several state-of-the-art predictors have been employed to evaluate the actual merits of the data collections as benchmark testing sets. All of the systems have demonstrated rational performance using the *H. sapiens *and *M. musculus *data sets constructed by us. Comparative study has also been conducted which reports the predictors' performance on three other existing benchmark data sets. Inconsistent behavior has been observed on some of the approaches. In particular, GENSCAN appears to perform inadequately when tested on two of the existing benchmark sets. This further justifies the need for some TIS benchmark collections of higher quality.

## Availability and Requirements

The data sets derived from four model organisms will be made available to the public through the following URL: 

## Authors' contributions

JZ created the data sets, designed and implemented the analyses, and drafted the manuscript. RA coordinated with the design and editing. DJD conceived of the study, contributed his expertise on molecular biology, participated in revising the analytical methods and helped with the editing. All authors read and approved the final manuscript.
